# Supplementary material for: Genome-wide identification, characterization and gene expression of BES1 transcription factor family in grapevine (Vitis vinifera L.)
Source: Sci Rep. 2023 Jan 5;13:240. doi: 10.1038/s41598-022-24407-y (PMC9816167; doi:10.1038/s41598-022-24407-y)
Supplement: Supplementary file 3 — Supplementary Information. [file 41598_2022_24407_MOESM3_ESM.zip › Vvi_Atr/Vitis_vinifera.PN40024.v4.dna_sm.toplevel.fa.vs.Amborella_trichopoda.AMTR1.0.dna_sm.toplevel.fa.html/Atr-AmTr_v1.0_scaffold00180.html]

|  |  |  |  |  |  |  |  |  |  |  |  |  |  |
| --- | --- | --- | --- | --- | --- | --- | --- | --- | --- | --- | --- | --- | --- |
| Duplication depth | Reference chromosome | Collinear blocks | | | | | | | | | | | |
| 0 | Atr-ERN12810 |  |  |  |  |  |  |  |  |  |  |  |  |
| 0 | Atr-ERN12811 |  |  |  |  |  |  |  |  |  |  |  |  |
| 0 | Atr-ERN12812 |  |  |  |  |  |  |  |  |  |  |  |  |
| 0 | Atr-ERN12813 |  |  |  |  |  |  |  |  |  |  |  |  |
| 0 | Atr-ERN12814 |  |  |  |  |  |  |  |  |  |  |  |  |
| 0 | Atr-ERN12815 |  |  |  |  |  |  |  |  |  |  |  |  |
| 0 | Atr-ERN12816 |  |  |  |  |  |  |  |  |  |  |  |  |
| 0 | Atr-ERN12817 |  |  |  |  |  |  |  |  |  |  |  |  |
| 0 | Atr-ERN12818 |  |  |  |  |  |  |  |  |  |  |  |  |
| 0 | Atr-ERN12819 |  |  |  |  |  |  |  |  |  |  |  |  |
| 0 | Atr-ERN12820 |  |  |  |  |  |  |  |  |  |  |  |  |
| 0 | Atr-ERN12821 |  |  |  |  |  |  |  |  |  |  |  |  |
| 0 | Atr-ERN12822 |  |  |  |  |  |  |  |  |  |  |  |  |
| 0 | Atr-ERN12823 |  |  |  |  |  |  |  |  |  |  |  |  |
| 0 | Atr-ERN12824 |  |  |  |  |  |  |  |  |  |  |  |  |
| 0 | Atr-ERN12825 |  |  |  |  |  |  |  |  |  |  |  |  |
| 0 | Atr-ERN12826 |  |  |  |  |  |  |  |  |  |  |  |  |
| 0 | Atr-ERN12827 |  |  |  |  |  |  |  |  |  |  |  |  |
| 0 | Atr-ERN12828 |  |  |  |  |  |  |  |  |  |  |  |  |
| 0 | Atr-ERN12829 |  |  |  |  |  |  |  |  |  |  |  |  |
| 0 | Atr-ERN12830 |  |  |  |  |  |  |  |  |  |  |  |  |
| 0 | Atr-ERN12831 |  |  |  |  |  |  |  |  |  |  |  |  |
| 0 | Atr-ERN12832 |  |  |  |  |  |  |  |  |  |  |  |  |
| 0 | Atr-ERN12833 |  |  |  |  |  |  |  |  |  |  |  |  |
| 0 | Atr-ERN12834 |  |  |  |  |  |  |  |  |  |  |  |  |
| 0 | Atr-ERN12835 |  |  |  |  |  |  |  |  |  |  |  |  |
| 0 | Atr-ERN12836 |  |  |  |  |  |  |  |  |  |  |  |  |
| 0 | Atr-ERN12837 |  |  |  |  |  |  |  |  |  |  |  |  |
| 0 | Atr-ERN12838 |  |  |  |  |  |  |  |  |  |  |  |  |
| 0 | Atr-ERN12839 |  |  |  |  |  |  |  |  |  |  |  |  |
| 0 | Atr-ERN12840 |  |  |  |  |  |  |  |  |  |  |  |  |
| 0 | Atr-ERN12841 |  |  |  |  |  |  |  |  |  |  |  |  |
